# ﻿A new study of *Nagrajomyces*: with two new species proposed and taxonomic status inferred by phylogenetic methods

**DOI:** 10.3897/mycokeys.93.93712

**Published:** 2022-10-25

**Authors:** Lan Zhuo, Mei-Jun Guo, Qiu-Tong Wang, Hao Zhou, Meike Piepenbring, Cheng-Lin Hou

**Affiliations:** 1 College of Life Science, Capital Normal University, Xisanhuanbeilu 105, Haidian, Beijing 100048, China; 2 Mycology Research Group, Faculty of Biological Sciences, Goethe University Frankfurt am Main, Max-von-Laue-Str. 13, D-60438 Frankfurt am Main, Germany; 3 Beijing Key Laboratory of Plant Gene Resources and Biotechnology for Carbon Reduction and Environmental Improvement, Capital Normal University, Xisanhuanbeilu 105, Haidian, Beijing 100048, China

**Keywords:** host specificity, *Nagrajomyces*, new taxa, phylogeny

## Abstract

*Nagrajomyces* (*incertae sedis*, *Ascomycota*) is a monotypic genus with a previously unknown systematic position. In this report, two new species are proposed, *Nagrajomycesfusiformis* and *Nagrajomyceslaojunshanensis.* These new taxa are proposed based on morphological characteristics evident via light microscopy and molecular data. Multi-locus phylogenetic analyses (ITS rDNA, nrLSU rDNA, *RPB2*, and *TEF1-α*) show that specimens recently collected in Yunnan Province, China are closely related to *Gnomoniaceae*. Both new species and known species were discovered repeatedly in their asexual developmental form exclusively on twigs of *Rhododendron* spp. (*Ericaceae*). This indicates a host specificity of *Nagrajomyces* spp. for species of *Rhododendron*.

## ﻿Introduction

*Gnomoniaceae* is a distinct family of *Diaporthales*, established by Winter (1886). The traditional classification of species in *Gnomoniaceae* was mainly based on morphological features, such as the morphology of ascomata and ascospores as well as the position of necks (Barr 1978; Monod 1983). Sogonov et al. (2008) used phylogenetic analyses of molecular sequence data of several genes (*TEF1-α*, nrLSU, and *RPB*2) to revise the concepts of leaf-inhabiting genera, and discovered that several traditional genera in *Gnomoniaceae* are polyphyletic. Phylogenetic analyses indicate that host specificity can be used to circumscribe genera and species of *Gnomoniaceae*. Based on phylogenetic analyses and morphological characteristics, Senanayake et al. (2017) described new taxa and excluded some genera from *Gnomoniaceae*. Since then, additional genera have been introduced for species observed in sexual developmental stages, as well as those that have only been observed as pycnidial asexual morphs, and rarely for species known in both sexual and asexual forms (Senanayake et al. 2018; Crous et al. 2019; Jiang et al. 2019; Minoshima et al. 2019; Yang et al. 2020).

Many species of *Gnomoniaceae* are important plant pathogens, such as *Apiognomoniaerrabunda* (Roberge ex Desm.) Höhn, which causes oak anthracnose (Sogonov et al. 2007), *Gnomoniopsisfructicola* (G. Arnaud) Sogonov, which causes strawberry stem rot (Maas 1998), and *Ophiognomonialeptostyla* (Fr.) Sogonov, which causes walnut anthracnose (Neely and Black 1976). Species of *Gnomoniaceae* can also have wide host ranges, with species on *Fagaceae*, *Onagraceae*, and *Rosaceae* being frequently infected. Species of Rhododendron (Ericaceae), which is the largest genus of woody plants in the northern hemisphere, are also known hosts of species of *Gnomoniaceae* (Monod 1983).

*Nagrajomyces* (*incertae sedis*, *Ascomycota*) is a monotypic genus based on *N.dictyosporus* Mel’nik (Mel’nik 1984; Nag Raj 1993). It was discovered in Russia, where it grows on twigs of *Rhododendronaureum* and develops stalked, unilocular, or plurilocular conidiomata as well as muriform conidia, and each conidium bears an apical appendage that is single, unbranched, attenuated, flexuous and can be more than 100 μm long (Mel’nik 1984; Nag Raj 1993).

In the present study, two new species were discovered on twigs of *Rhododendron* spp. in Yunnan and assigned to the genus *Nagrajomyces* based on morphological characteristics, habitat, and host. Phylogenetic analysis revealed that the proposed *Nagrajomyces* species belong to *Gnomoniaceae*.

## ﻿Materials and methods

### ﻿Specimen collections and isolation

Fieldwork for the discovery of fungi was conducted during June 2021 in Yunnan Province, China. Fresh pycnidia were repeatedly discovered and collected on twigs of *Rhododendron* spp. Twigs with conidiomata were packed in paper bags and transported to the laboratory for morphological tests. Conidiomata were cut off in the laboratory using a razor blade, wrapped in paper packets, disinfected with 75% ethanol for 10 s, then 10% sodium hypochlorite for 2 min 30 s, and rinsed with distilled water three times. After absorbing the water with sterile filter paper, the conidiomata were transferred to potato dextrose agar (PDA) plates (Jiang et al. 2021) then incubated at 25 °C to obtain cultures. Dry specimens were deposited at the China Forest Biodiversity Museum of the Chinese Academy of Forestry (CAF; http://museum.caf.ac.cn) and the Herbarium of the College of Life Science, Capital Normal University (BJTC; http://smkxxy.cnu.edu.cn). Ex-type living cultures were deposited at the China Forestry Culture Collection Center (CFCC; http://cfcc.caf.ac.cn/).

### ﻿Morphological analysis

Conidiomata were photographed and cut by hand using a razor blade under a Nikon SMZ-1000 stereomicroscope (Japan). Morphological characteristics of conidiomata, conidiophores, and conidia were photographed and measured with an Olympus EX-51 upright microscope (Japan), and for each structure at least 20 measurements were made. Color values were taken from ColorHexa (https://www.colorhexa.com/).

### ﻿DNA extraction, polymerase chain reaction amplification, and phylogeny

Genomic DNA was extracted from specimens and cultures via the M5 Plant Genomic DNA Kit (Mei5 Biotechnology Co., Ltd., China) in accordance with the manufacturer’s instructions. Table 1 summarizes the primers used to obtain sequence data for ITS rDNA, nrLSU rDNA, *RPB2*, and *TEF1-α*, and the polymerase chain reaction (PCR) amplification protocols. PCR products were analyzed in 1% electrophoretic agarose gel with a 200-bp DNA ladder, purified, and sequenced by Beijing Zhongke Xilin Biotechnology Co., Ltd. (Beijing, China). SeqMan was used to align the sequences obtained by forward and reverse primers to obtain a consensus sequence. A partition homogeneity test was performed to determine the congruence of the four datasets (Farris et al. 1994). Sequences for phylogenetic analyses were selected based on Yang et al. (2020), supplemented by sequences of *Apiosporopsiscarpinea* (Fr.) Mariani, *Apiosporopsis* sp., *Juglanconisjuglandina* (Kunze) Voglmayr & Jaklitsch, *Juglanconisoblonga* (Berk.) Voglmayr & Jaklitsch, and *Melanconismarginalis* (Peck) Wehm. from Senanayake et al. (2018) used as outgroup taxa. All sequences used in this study are listed in Table 2. Subsequent alignments were generated with online MAFFT tools (https://www.ebi.ac.uk/Tools/msa/mafft/) and edited with Gblocks 0.91b (http://molevol.cmima.csic.es/castresana/Gblocks_server.html). The maximum likelihood (ML) tree was constructed using RAxML version 8.2.12 (Stamatakis et al. 2005; Stamatakis 2006; Stamatakis 2014) with GTRGAMMA model and 1000 bootstrap iterations. The multi-locus Bayesian Inference (BI) tree was built by MrBayes version 3.2.6 (Ronquist and Huelsenbeck 2003). Models of nucleotide substitution for each gene used in the Bayesian analysis were determined by MrModeltest v.2.3 (Nylander 2004). Analyses of four simultaneous Markov Chain Monte Carlo (MCMC) chains were run for 100,000,000 generations, and other operational methods were applied used as described by Guo et al. (2021). The maximum parsimony (MP) tree was constructed using PAUP version 4.0 beta 10 (Swofford 2003) with 1000 random sequence additions, 1000 maxtrees were obtained, and bootstrap analysis was conducted based on 1000 replicates, with 10 replicates of random stepwise additions of taxa. For further details see Guo et al. (2021). Trees were viewed via Treeview (Page 1996).

**Table 1.** Primer information and PCR amplification protocols.

Gene	Primer pairs	Reference	Amplification conditions
ITS rDNA	ITS1F/ITS4	White et al. (1990); Gardes and Bruns (1993)	Phillips et al. (2008)
LSU rDNA	LR0R/LR5	Vilgalys and Hester (1990); Rehner and Samuels (1994)	Phillips et al. (2008)
*TEF1-α*	EF1-728F/EF1-986R	Carbone and Kohn (1999)	Glass and Donaldson (1995)
*RPB2*	fRPB2-5F/fRPB2-7cR	Liu et al. (1999)	Liu et al. (1999)

**Table 2.** Sequences used in phylogenetic analyses. References to sequences generated in the present study are emphasized in bold.

Taxa	Voucher	ITS rDNA	LSU rDNA	*RPB2*	*TEF1*-α	References
*Alneciumauctum*	CBS 124263	KF570154	KF570154	KF570170	KF570200	Voglmayr and Jaklitsch (2014)
*Ambarignomoniapetiolorum*	CBS 116866	EU199193	AY818963	EU199151	–	Mejía et al. (2008)
*Ambarignomoniapetiolorum*	CBS 121227	EU254748	EU255070	EU219307	EU221898	Mejía et al. (2008)
*Amphiporthetiliae*	CBS 119289	EU199178	EU199122	EU199137	–	Mejía et al. (2008)
*Apiognomoniaerrabunda*	AR 2813	DQ313525	–	DQ862014	DQ313565	Sogonov et al. (2007)
*Apiognomoniaveneta*	MFLUCC 16-1193	MF190114	MF190056	–	–	Senanayake et al. (2017)
*Apioplagiostomapopuli*	858501	KP637024	–	–	–	Wijekoon et al. (2021)
*Apiosporopsiscarpinea*	CBS 771.79	–	AF277130	–	–	Zhang and Blackwell (2001)
*Apiosporopsis* sp.	Masuya 11Af2-1	–	AB669034	–	–	Osono and Masuya (2012)
*Asteromaalneum*	CBS 109840	EU167609	EU167609	–	–	Simon et al. (2009)
*Asteroma* sp.	Masuya 8Ah9-1	–	AB669035	–	–	Osono and Masuya (2012)
*Cryptodiaportheacerina*	AR 3822	EU254755	EU255075	EU219253	EU221879	Sogonov et al. (2008)
*Cryptodiaportheaubertii*	CBS 114196	KX929767	KX929803	KX929838	KX929732	Meyer et al. (2017)
*Cryptosporellahypodermia*	CBS 116866	EU199181	AF408346	EU199140	–	Mejía et al. (2008)
*Ditopellabiseptata*	MFLU 15-2661	MF190147	MF190091	MF377616	–	Senanayake et al. (2017)
*Ditopelladitopa*	CBS 109748	DQ323526	EU199126	EU199145	–	Mejía et al. (2008)
*Ditopellopsis* sp.	CBS 121471	EU254763	EU255088	EU219254	EU221936	Sogonov et al. (2008)
*Flavignomoniarhoigena*	CFCC 53118	MK432674	MK429917	MK578102	–	Jiang et al. (2019)
*Flavignomoniarhoigena*	CFCC 53119	MK432675	MK429918	MK578103	–	Jiang et al. (2019)
*Gnomoniagnomon*	CBS 199.53	DQ491518	AF408361	EU219295	EU221885	Sogonov et al. (2008)
*Gnomoniagnomon*	CBS 829.79	AY818957	AY818964	–	EU221905	Sogonov et al. (2005)
*Gnomoniellamicrospora*	BPI 877571	EU254765	–	–	–	Sogonov et al. (2008)
*Gnomoniopsisalderdunensis*	CBS 125680	GU320825	–	–	–	Walker et al. (2010)
*Gnomoniopsischamaemori*	CBS 803.79	EU254808	EU255107	–	–	Sogonov et al. (2008)
*Gnomoniopsisracemula*	AR 3892	EU254841	EU255122	EU219241	EU221889	Sogonov et al. (2008)
*Juglanconisjuglandina*	WU 35960	KY427145	KY427145	KY427195	KY427214	Voglmayr et al. (2017)
*Juglanconisoblonga*	TFM FPH 2623	KY427153	KY427153	KY427203	KY427222	Voglmayr et al. (2017)
*Mamianiellacoryli*	BPI 877578	EU254862	–	–	–	Sogonov et al. (2008)
*Marsupiomycesepidermoidea*	MFLU 15-2921	–	MF190058	–	–	Senanayake et al. (2017)
*Marsupiomycesquercina*	MFLUCC 13-0664	MF190116	MF190061	–	–	Senanayake et al. (2017)
*Melanconismarginalis*	BPI 748234	–	–	EU219299	EU221886	Sogonov et al. (2008)
*Melanconismarginalis*	BPI 748446	EU199197	AF408373	EU219301	EU221991	Sogonov et al. (2008)
*Neognomoniopsisquercina*	CBS 145575	MK876399	MK876440	–	–	Crous et al. (2019)
***Nagrajomycesfusiformis***	**CAF 800050**	**OP473599**	**OP473595**	**OP484756**	**OP484760**	**This study**
***Nagrajomycesfusiformis***	**BJTC 1773**	**OP473602**	**OP473598**	–	**OP484763**	**This study**
***Nagrajomyceslaojunshanensis***	**CFCC 58177**	**OP456161**	**OP473594**	**OP484755**	**OP484759**	**This study**
***Nagrajomyceslaojunshanensis***	**CAF 800049**	**OP473600**	**OP473596**	**OP484757**	**OP484761**	**This study**
***Nagrajomyceslaojunshanensis***	**BJTC 1849**	**OP473601**	**OP473597**	**OP484758**	**OP484762**	**This study**
*Occultocarponailaoshanense*	LCM 524.01	JF779849	JF779853	JF779856	–	Mejía et al. (2011)
*Occultocarponailaoshanense*	LCM 522.01	JF779848	JF779852	JF779857	JF779862	Mejía et al. (2011)
*Ophiognomoniamelanostyla*	LCM 389.01	JF779850	JF779854	JF779858	–	Mejía et al. (2011)
*Ophiognomoniavasiljevae*	AR 4298	EU254977	EU255162	EU219331	EU221999	Sogonov et al. (2008)
*Phragmoportheconformis*	AR 3632	–	AF408377	–	–	Castlebury et al. (2002)
*Plagiostomaaesculi*	AR 3640	EU254994	EU255164	EU219269	–	Sogonov et al. (2008)
*Plagiostomarhododendri*	CBS 847.79	EU255044	EU255187	EU219272	–	Sogonov et al. (2008)
*Pleurocerasoregonense*	AR 4333	EU255060	EU255196	EU219313	EU221931	Sogonov et al. (2008)
*Pleuroceraspleurostylum*	CBS 906.79	EU255061	EU255197	EU219311	EU221962	Sogonov et al. (2008)
*Sirococcusconigenus*	BPI 871248	EU199201	EU199134	EU199157	–	Mejía et al. (2008)
*Sirococcuspiceicola*	BPI 871166	EU199202	EU199135	EU199158	–	Mejía et al. (2008)
*Sirococcustsugae*	BPI 871167	EU199203	EU199136	EU199159	–	Mejía et al. (2008)
*Sirococcustsugae*	AR 4010	EF512478	EU255207	EU219289	EU221928	Sogonov et al. (2008)
*Tenuignomoniastyracis*	BPI 892786	–	LC379289	LC379295	LC379283	Minoshima et al. (2019)
*Tenuignomoniastyracis*	BPI 892785	–	LC379288	LC379294	LC379282	Minoshima et al. (2019)
*Valsalnicolaoxystoma*	AR 5137	JX519561	–	–	–	Crous et al. (2012)
*Valsalnicolaoxystoma*	AR 4833	JX519559	JX519563	–	–	Crous et al. (2012)

## ﻿Results

### ﻿Phylogenetic analysis

**Figure 1.**
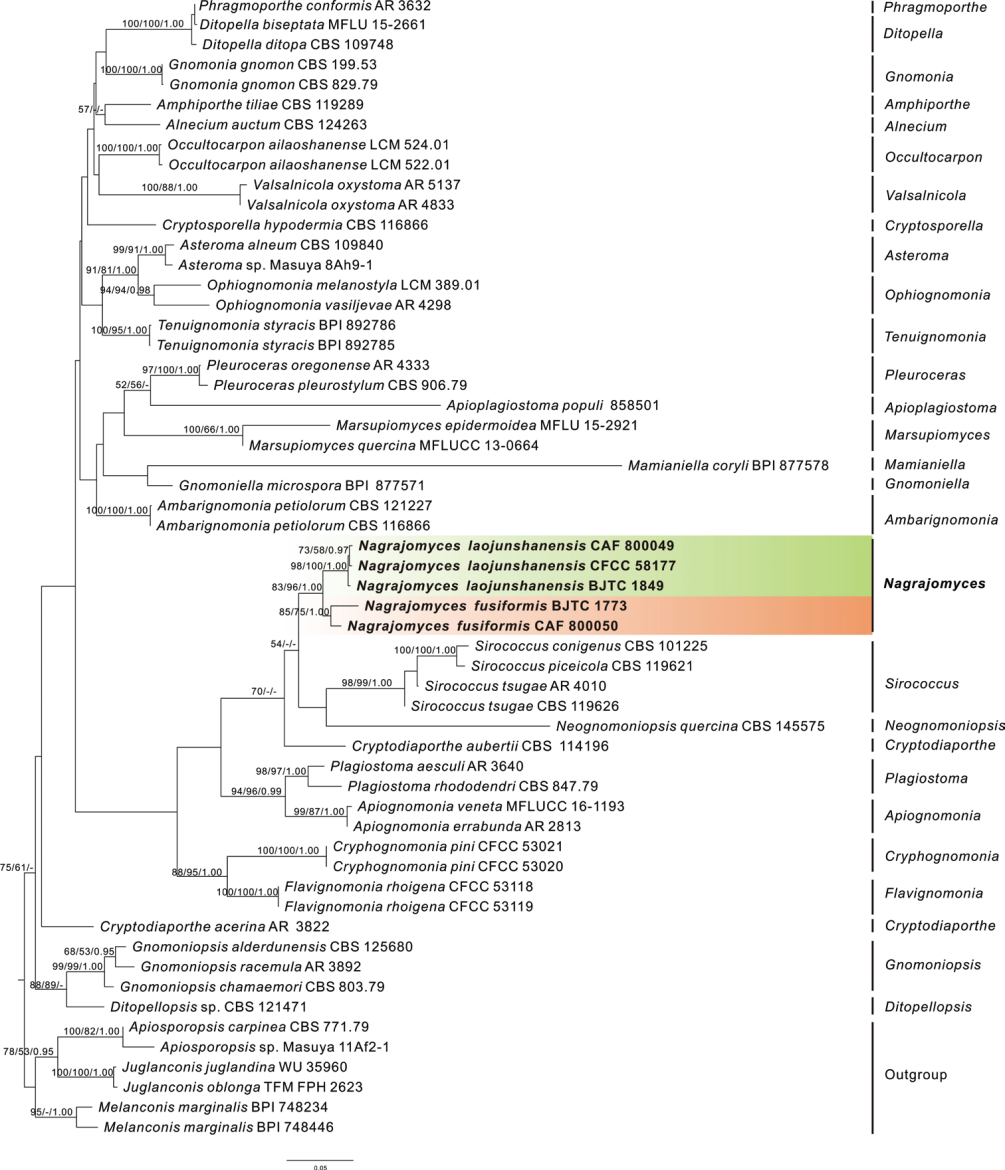
Phylogenetic tree based on an ML analysis of combined ITS rDNA, nrLSU rDNA, *RPB2*, and *TEF1-α* sequences of species of Gnomoniaceae. Bootstrap support values for RAxML and maximum parsimony above 50% and Bayesian posterior probability values above 0.95 are shown at the nodes. The tree is rooted with sequences of *Apiosporopsiscarpinea*, *Apiosporopsis* sp., *Juglanconisjuglandina*, *J.oblonga*, and *Melanconismarginalis*. References to new sequences are in bold, and the names of the two new species are highlighted by colors.

Multi-locus phylogenetic analyses of species of Gnomoniaceae (Diaporthales) include sequences of 51 ingroup taxa and sequences of an outgroup formed by *Apiosporopsiscarpinea*, *Apiosporopsis* sp., *Juglanconisjuglandina*, *J.oblonga*, and *Melanconismarginalis* (Fig. 1). The multi-locus dataset (ITS rDNA, LSU rDNA, *RPB2* and *TEF1-α*) comprises 2875 characters, of which 945 are parsimony-informative, 200 are parsimony-uninformative and 1730 are constant. Maximum parsimony analysis of sequences resulted in one most parsimonious tree with a length (TL) of 3730 steps, a consistency index (CI) of 0.463, a retention index (RI) of 0.690, and a homoplasy index (HI) of 0.537. Bayesian and maximum likelihood trees exhibited topologies similar to this parsimony tree.

The topology of the phylogenetic tree obtained in the current study was similar to the topology presented by Yang et al. (2020). Nineteen sequences of five specimens recently collected on *Rhododendron* spp. in China form a clade with high support values. This clade is sister to sequences of species of *Siroccocus* and *Neognomoniopsis*, but with poor support values. The newly discovered clade is divided into two small subclades labeled *Nagrajomycesfusiformis* and *N.laojunshanensis*.

### ﻿Taxonomy

#### ﻿ Nagrajomyces fusiformis

Taxon classificationFungi

C. L. Hou & L. Zhuo sp. nov.

B99EECB6-1A78-5CC3-BCEB-22A0E94A855E

845666

Figs 2 , 3

##### Etymology.

The epithet *fusiformis* refers to fusoid conidia.

##### Type.

China, Yunnan province, Lijiang, Yulong, 26°40'55"N, 99°54'01"E, alt. 2762 m, on dying twigs of *Rhododendronvellereum* Hutch. ex Tagg., 20 June 2021, coll. C.L. Hou, M.J. Guo, H. Zhou (holotype CAF 800050).

##### Diagnosis.

This new species differs from *N.dictyosporus* and *N.laojunshanensis* by fusoid to elongate-fusoid conidia with pointed ends, usually 1-septate and smaller.

**Figure 2.**
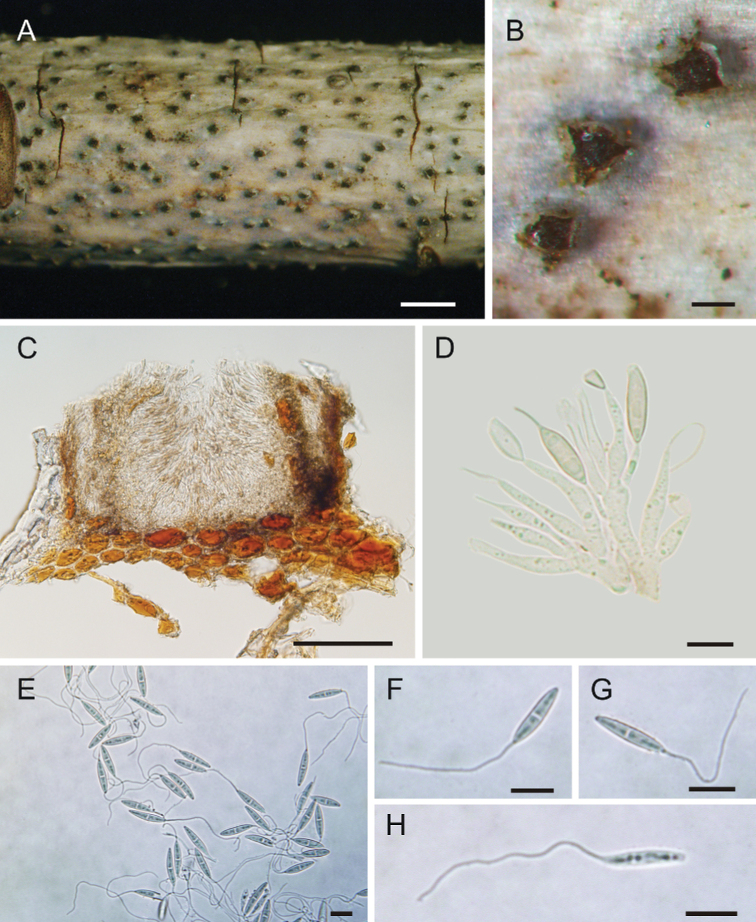
Micrographs of *Nagrajomycesfusiformis* (holotype CAF 800050) on twigs of *Rhododendronvellereum***A, B** conidiomata on a dying twig **C** vertical section of a conidioma **D** conidiophores and conidia at diverse developmental stages **E–H** conidia with appendages. Scale bars: 2 mm (**A**); 200 µm (**B**); 100 µm (**C**); 10 µm (**D–H**).

**Figure 3.**
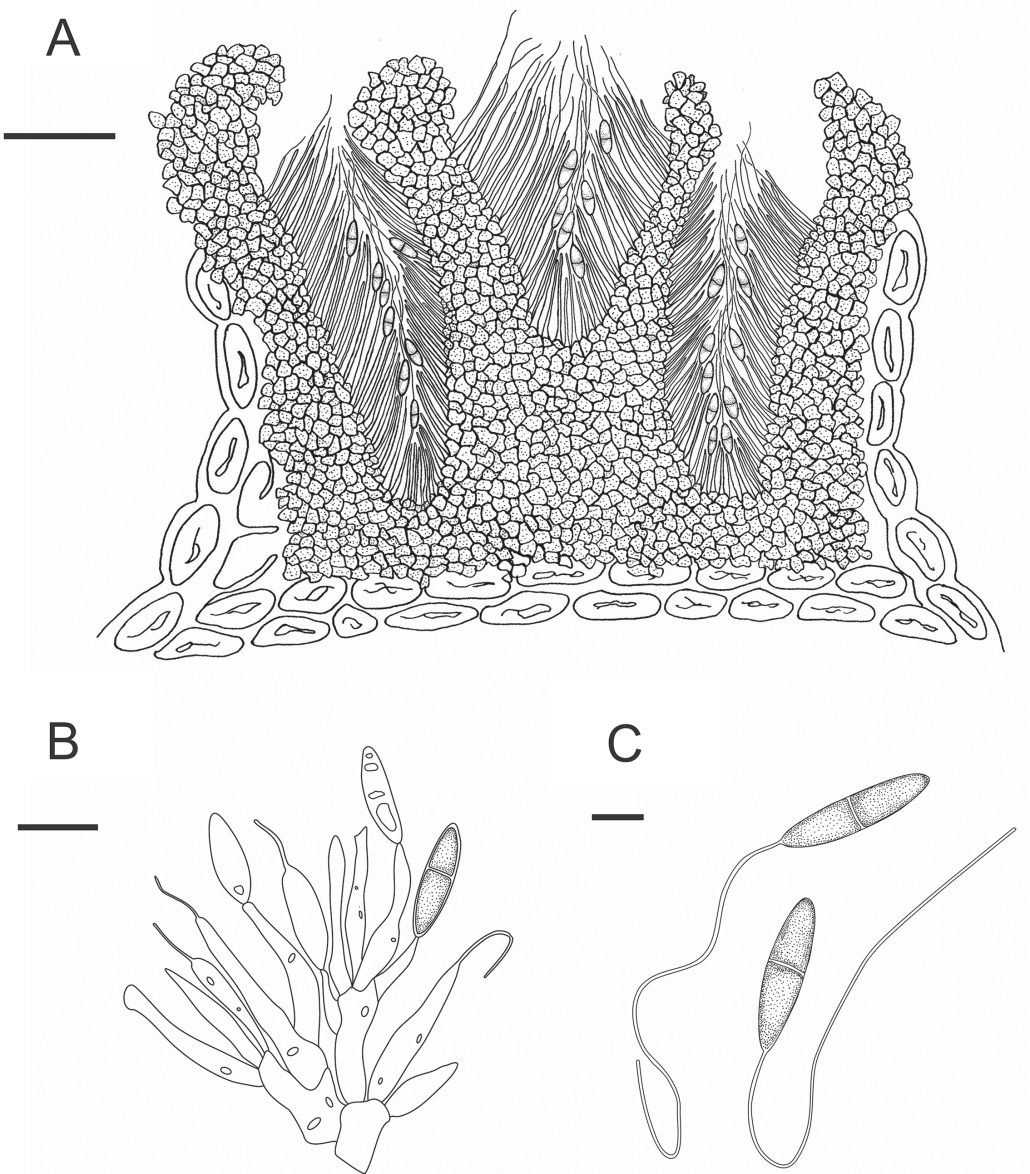
*Nagrajomycesfusiformis* (holotype CAF 800050) **A** vertical section of a conidioma **B** conidiophores and conidia **C** conidia with appendages. Scale bars: 100 µm (**A**); 10 µm (**B**); 5 µm (**C**).

##### Description.

**Figure 4.**
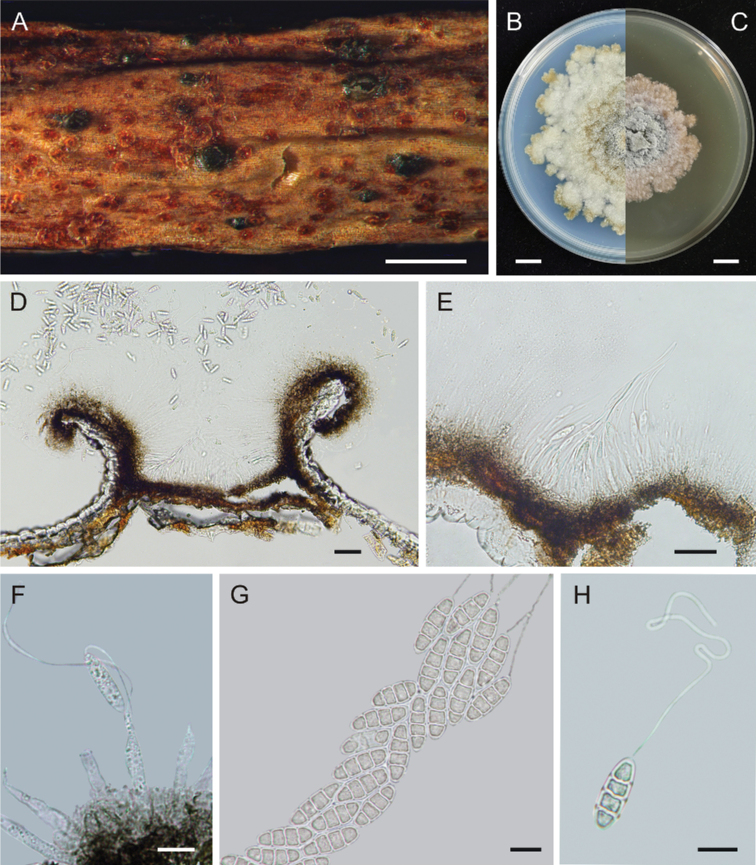
Micrographs of *Nagrajomyceslaojunshanensis* on *Rhododendroncinnabarinum* (holotype CAF 800049) **A** conidiomata on a living twig **B** ex-type culture (CFCC 58177) on PDA after 30 days, seen from above **C** ex-type culture (CFCC 58177) on MEA after 30 days, seen from above **D** vertical section of a conidioma **E, F** conidiophores and conidia **G** conidia forming a cirrus **H** conidium with appendage. Scale bars: 1 mm (**A**); 1 cm (**B, C**); 100 µm (**D, E**); 10 µm (**F–H**).

***Conidiomata*** solitary, pycnidial, irregularly plurilocular, subepidermal in origin, immersed at first, then becoming erumpent through the periderm of the host, 545–554 μm diameter, 520–546 μm high, peridium dark brown, 47.0–67.5 μm thick. ***Conidiophores*** ampulliform, smooth, hyaline, multiguttulate, 12–29 × 2.0–3.5 μm (x̄ = 19 × 3 μm, *n* = 20). ***Conidia*** fusoid to elongate-fusoid, 1-septate, cells equal, smooth, hyaline to pale brown, 13.5–19.0 × 3–4 μm (x̄ = 16.5 × 3.5 μm, *n* = 20), with a whip‑like appendage at the tip of each conidium, 30–77 μm (x̄ = 51 μm, *n* = 20) in length (Fig. 4). ***Sexual morph*** not observed.

##### Additional specimen examined.

China, Yunnan Province, Lijiang, Laojunshan, 26°37'56"N, 99°43'30"E, alt. 3873 m, on dying twigs of *Rhododendronvellereum*, 20 June 2021, coll. C.L. Hou, M.J. Guo, H. Zhou (BJTC 1773).

##### Notes.

*Nagragomycesfusiformis* differs from other species of *Nagrajomyces* by narrower and 1-septate conidia.

#### ﻿ Nagrajomyces laojunshanensis

Taxon classificationFungi

C. L. Hou & L. Zhuo sp. nov.

AEA1A9EF-91C8-51AF-AC56-199595378F03

845665

Figs 4 , 5

##### Etymology.

The epithet *laojunshanensis* refers to the location where the type specimen was collected.

##### Type.

China, Yunnan Province, Lijiang, Laojunshan, 26°39'44"N, 99°46'58"E, alt. 2910 m, on living twigs of *Rhododendroncinnabarinum* Hook. f., 20 June 2021, coll. C.L. Hou, M.J. Guo, H. Zhou (holotype CAF 800049). Ex-type culture CFCC 58177.

##### Diagnosis.

This new species differs from *N.fusiformis* by conidia that are elongate-elliptical, blunter at both ends, and usually 3-septate and larger. *Nagrajomyceslaojunshanensis* differs from *N.dictyosporus* by conidiomata that are unilocular and without stalks.

##### Description.

***Conidiomata*** solitary, pycnidial, unilocular, subglobose to ellipsoidal, subepidermal in origin, immersed at first, then becoming erumpent, 218–406 μm wide, 188–275 μm high, peridia black, 37–43 μm thick, opening irregularly in the upper part, with faint yellow content. ***Conidiophores*** ampulliform, smooth, hyaline, multiguttulate, 16.0–25.5 × 2–4 μm (x̄ = 21 × 3 μm, *n* = 20). ***Conidia*** elongate-elliptical, 1–3-septate, mostly 3-septate, smooth, hyaline, 18–23 × 5.5–7.0 μm (x̄ = 19.5 × 6.5 μm, *n* = 20), with a long, whip-like appendage at the tip of each conidium, 70–200 μm (x̄ = 143.5 μm, *n* = 20) in length. ***Sexual morph*** not observed.

##### Culture characteristics.

***Cultures*** (ex-type CFCC 58177) on PDA 8 cm diameter after 1 month, with irregular margins, sparse aerial mycelium, colonies with whitish margins, with center turning black olive (#3b3c36) with increasing age. On MEA, 5.7 cm diameter after 1 month, with irregular margins, colonies with beaver (#9f8170) -colored margins, with center turning black olive (#3b3c36) with increasing age. ***Conidia*** not observed.

##### Additional specimen examined.

China, Yunnan province, Kunming, Luquan, Jiaozixueshan, 26°05'04"N, 102°50'54"E, alt. 3823 m, on living twigs of *Rhododendroncinnabarinum* Hook. f., 23 June 2021, coll. C.L. Hou, M.J. Guo, H. Zhou, (BJTC 1849).

##### Notes.

*Nagrajomyceslaojunshanensis* differs from *N.dictyosporus* by conidia that are colorless and conidiomata that are without stalks. *Nagrajomyceslaojunshanensis* differs from *N.fusiformis* by elongate-elliptical conidia with blunter ends, which are longer (18–23 μm vs. 13–19 μm) and wider (5.7–7.0 μm vs. 2.8–3.7 μm). Conidia of *N.laojunshanensis* are mostly 3-septate, whereas those of *N.fusiformis* are 1-septate. Molecular sequence data confirm the presence of two distinct species.

**Figure 5.**
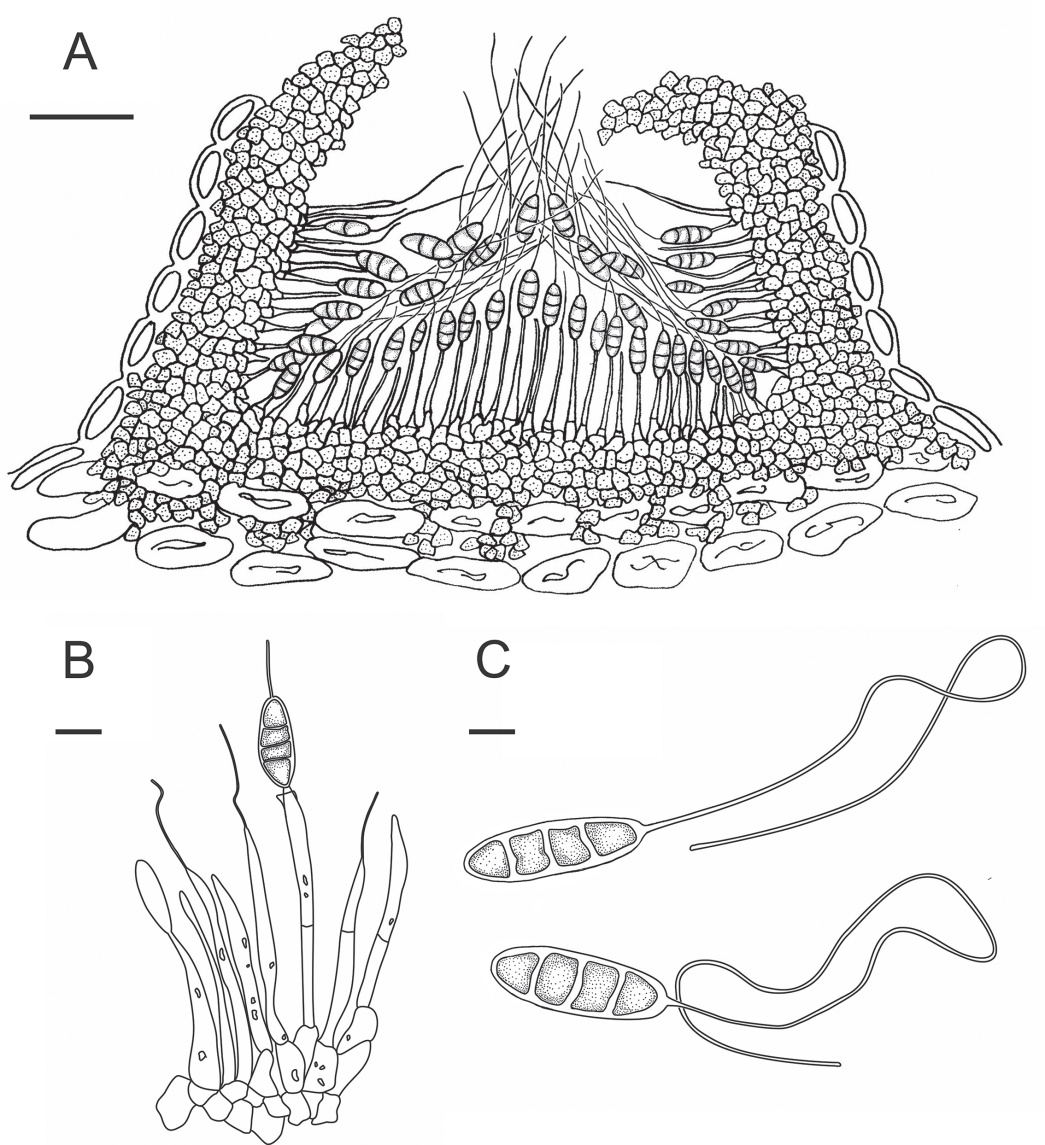
*Nagrajomyceslaojunshanensis* (holotype CAF 800049) **A** vertical section of a conidioma **B** conidiophores and conidia **C** conidia with appendages. Scale bars: 50 µm (**A**); 10 µm (**B**); 5 µm (**C**).

## ﻿Discussion

Morphologically, the most distinctive features of the new species of *Nagrajomyces* are septate conidia with long, single, apical appendages. The presence of this structure distinguishes them from all anamorphic genera known to belong to *Gnomoniaceae*. Both new species proposed in the present study and the known species *N.dictyosporus* inhabit twigs of *Rhododendron*. In spite of the absence of molecular data for the type species of *Nagrajomyces*, these two new species are accommodated in *Nagrajomyces* based on significant morphological features (distinctive conidia) and identical ecology.

Many coelomycetous genera have conidia with appendages (Nag Raj 1993), and some of them share morphological characteristics with the new species proposed in this study. For example, species of *Uniseta* and *Urohendersonia* have septate conidia and a long apical appendage attached to each conidium. *Uniseta* is a monotypic genus typified by *U.flagellifera* (Ellis & Everh.) Ciccar. (Ciccarone 1947). Nag Raj (1974, 1993) mentioned that *U.flagellifera* has a sexual morph called *Cryptodiaporthecomptoniae* (Schwein.) Barr (Barr 1991, syn. C.aubertiivar.comptoniae (Schwein.) Wehm.) that is considered a synonym of *Cryptodiaportheaubertii* (Westend.) Wehm. (Wehmeyer 1933). Conidia of this species are two-celled, hyaline, relatively inequilateral or curved, and bear a long flagellate appendage at one end (Wehmeyer 1933). *Cryptodiaportheacerina* J. Reid & Cain and *C.aubertii* are included in the phylogenetic tree (Fig. 1) and located distant from the new species proposed herein. Furthermore, *U.flagellifera* differs from the new species proposed here by an asexual morph growing on branches of *Comptoniaasplenifolia* damaged by fire (Ellis and Everhart 1889), while *Nagrajomyces* spp. develop on twigs of *Rhododendron*. Because of these differences, we consider the genus *Uniseta* to be separate from the genus *Nagrajomyces*.

Spegazzini (1902) introduced *Urohendersonia* Speg. with *Ur.platensis* Speg. as the type species. Nag Raj (1993) listed only five species in this genus, including the type species. *Urohendersonia* spp. differ from *Nagrajomyces* spp. by having globose to subglobose conidiomata immersed in host tissues, and yellowish brown to brown conidia each with an extracellular gelatinous appendage, and their host species (Nag Raj 1993). *Urohendersonia* spp. occur on diverse host species and various substrates of host, such as on leaves of *Erythrina* sp., *Manihotcarthagenensis*, *Pongamiapinnnata*, *Stipaspartea*, or in the rhizospheres of *Acervapersica* and *Dactylocteniumaegyptium* (Nag Raj 1993; Wijayawardene et al. 2016). Unfortunately, there are no molecular sequence data available for any species within those genera.

In the phylogenetic analysis presented herein, the two new species, *N.fusiformis* and *N.laojunshanensis* form a clade with high support values, which is separate from other species of *Gnomoniaceae* represented by sequence data in GenBank. These two new species described in this study fill gaps in the molecular data of *Nagrajomyces* and also enable the taxonomic status of the new species to be determined.

A total of 38 genera are currently included in the family *Gnomoniaceae* based on morphological and molecular analyses (Senanayake et al. 2018; Crous et al. 2019; Jiang et al. 2019; Minoshima et al. 2019; Yang et al. 2020). Sexual morphs have been described for all but four; *Asteroma*, *Flavignomonia*, *Millerburtonia*, and *Sirococcus*. *Sirococcus* spp. are closely related to the new species described herein, whereas phylogenetic data indicate that the other three genera are distantly related to *Nagrajomyces* spp. *Asteroma* spp. have cylindrical to fusiform, acicular or broadly fusiform conidia (Senanayake et al. 2018). Conidia of *Flavignomonia* are cylindrical to oblong (Jiang et al. 2019), and conidia of *Millerburtonia* are filiform and aciculate (Ciferri 1951).

In addition to morphological characteristics and molecular sequence data, host ranges are often useful to delineate genera and species of *Gnomoniaceae* (Sogonov et al. 2008). Species of *Gnomonia*, for example, are generally associated with host plants in the *Betulaceae* family, mostly belonging to the subfamily *Coryloideae* (Sogonov et al. 2008). The two new species identified in the present study, and the known species, all develop on twigs of *Rhododendron* spp. indicating that they are specialized with respect to this host. The differences in conidiomatal structure could be explained by differences in host epidermal features or maturity. Two species of *Gnomoniaceae* are known to inhabit *Rhododendron* spp. *Plagiostomarhododendri* (Auersw.) Sogonov was reported on dry twigs and inflorescences of *Rhododendronhirsutum* L., and occasionally on dead leaves of *R.ferrugineum* L. (Monod, 1983). Only the sexual form of this species has been described, and phylogenetic analysis places it somewhat distant to species of *Nagrajomyces* (Fig. 1). The second species is *Gnomonia* sp., reported on rotten leaves of *R.ferrugineum* (Rehm 1906). This species lacks a specific morphological description.

*Rhododendron* is the largest genus of woody plants in the northern hemisphere, and its species diversity is highest in the Himalaya-Hengduan Mountains and Southeast Asia (Chamberlain et al. 1996; Shrestha et al. 2018). Considering the host preference of *Gnomoniaceae* species and the biodiversity of *Rhododendron* worldwide, additional *Gnomoniaceae* species are expected to exist on these plants.

## Supplementary Material

XML Treatment for Nagrajomyces fusiformis

XML Treatment for Nagrajomyces laojunshanensis
